# Nuclear PI3K signaling in cell growth and tumorigenesis

**DOI:** 10.3389/fcell.2015.00024

**Published:** 2015-04-13

**Authors:** William J. Davis, Peter Z. Lehmann, Weimin Li

**Affiliations:** College of Medical Sciences, Washington State UniversitySpokane, WA, USA

**Keywords:** nuclear signaling, PI3K/Akt/mTOR, cell growth, tumorigenesis, ribosome biogenesis, cell survival, DNA damage, mRNA processing and export

## Abstract

The PI3K/Akt signaling pathway is a major driving force in a variety of cellular functions. Dysregulation of this pathway has been implicated in many human diseases including cancer. While the activity of the cytoplasmic PI3K/Akt pathway has been extensively studied, the functions of these molecules and their effector proteins within the nucleus are poorly understood. Harboring key cellular processes such as DNA replication and repair as well as nascent messenger RNA transcription, the nucleus provides a unique compartmental environment for protein–protein and protein–DNA/RNA interactions required for cell survival, growth, and proliferation. Here we summarize recent advances made toward elucidating the nuclear PI3K/Akt signaling cascade and its key components within the nucleus as they pertain to cell growth and tumorigenesis. This review covers the spatial and temporal localization of the major nuclear kinases having PI3K activities and the counteracting phosphatases as well as the role of nuclear PI3K/Akt signaling in mRNA processing and exportation, DNA replication and repair, ribosome biogenesis, cell survival, and tumorigenesis.

## Introduction

In the late 1970s and early 1980s, the existence of a nuclear phosphatidylinositol (PtdIns) cycle was proposed (Manzoli et al., 1978, 1982). PtdIns consist of an inositol ring and two acyl chains. The inositol ring moiety can be phosphorylated at different positions by PtdIns kinases to generate phosphoinositides (PIs), which can serve as primary or secondary signaling messengers. Breakthroughs in the field were made when PtdIns 4-phosphate (PI4P), PtdIns 4,5-bisphosphate [PI(4,5)P_2_], and the relevant kinases were identified within the nucleus and found to be regulated independent of the cytosolic pools (Cocco et al., 1987; Divecha et al., 1991; Boronenkov et al., 1998). Further work identified PI(3,4,5)P_3_, its kinases and phosphatases in the nucleus (Lindsay et al., 2006; Song et al., 2012; Elong Edimo et al., 2013). Interestingly, the localization and activities of these nuclear PIs and their kinases are not associated with any currently known membranous structures, implying distinct nuclear functions separate from those in the cytoplasm. What both the nuclear and the cytoplasmic compartments do have in common for the PtdIns 3-kinase (PI3K)-mediated cascade is the kinase- and phosphatase-dependent and -independent regulation of downstream biological effects.

**Figure 1:**
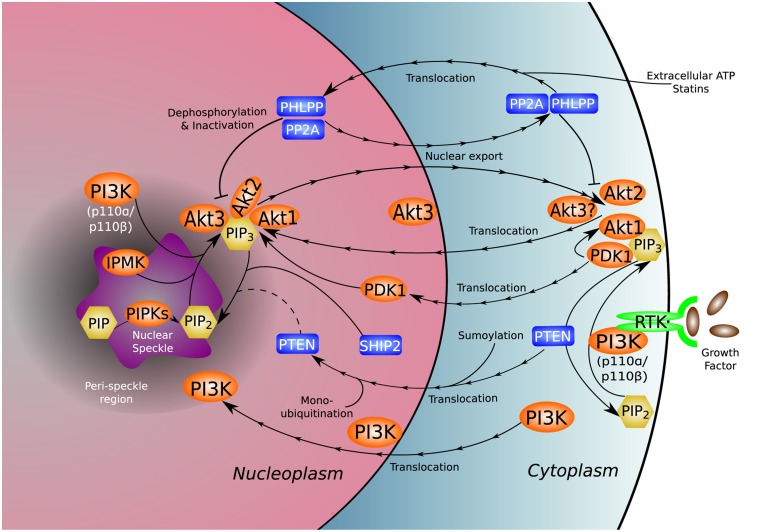
**Nuclear distribution of the PI3K pathway**. PI3K and many of its associated kinases (orange), phosphatases (blue), and phosphoinositides (yellow) reside natively in or translocate to the nucleus and nuclear sub-compartments, adding complexity to the established functional repertoire of the canonical PI3K pathway. The unique nuclear environment gives rise to non-canonical functions and molecular interactions not present in the cytoplasm. The uncertain role of nuclear PTEN in dephosphorylation of nuclear PI(3,4,5)P_3_ is denoted by a dotted line.

The PI3K pathway mediates a wide range of cellular processes including cell survival, migration, division, differentiation, and proliferation. Mutations and dysregulation of key signal-relaying enzymes in this pathway are frequently found in a variety of human pathological conditions (Vanhaesebroeck et al., 2010). While this review focuses on the relationship of PI3K to cancer, the nuclear PI3K pathway is relevant in other human diseases such as cardiovascular disorders. The nuclear localization and translocation of specific PI3K and v-akt murine thymoma viral oncogene homolog (Akt)/protein kinase B (PKB) isoforms under different signaling events expand our current understanding of the functions of these molecules (Figure 1). The combinational insights provided by studies in both the cytosolic and nuclear compartments will help construct a comprehensive picture of the biological functions regulated by these enzymes.

## Spatial and temporal positioning of nuclear PI3K activity

PI(4,5)P_2_, the predominant precursor for PI(3,4,5)P_3_ generation, was observed localizing to nuclear speckles (Boronenkov et al., 1998). Disruption of the PI(4,5)P_2_ speckle pool, for example, by depletion of the PDZ-domain-containing scaffolding protein syntenin implicated in melanoma cell growth and differentiation (Lin et al., 1998; Beekman and Coffer, 2008), impairs cell survival and division in a signaling-dependent manner (Mortier et al., 2005; Beekman and Coffer, 2008). In contrast to the localization of PI(4,5)P_2_, PI(3,4,5)P_3_ was found in the nuclear matrix around nuclear speckles (Lindsay et al., 2006). These studies suggest that the two different nuclear phosphoinositide pools may not overlap with each other. Perhaps the PI(3,4,5)P_3_ generated from PI(4,5)P_2_ or other PI resources are targeted to the nuclear matrix and other nuclear spots, such as the inner nuclear leaflet and protein complexes, for protein docking or/and activation. It is not clear whether this is a cell type-specific distribution or a cell signaling, state-dependent change. It would be informative to see the subnuclear localization of PI(4,5)P_2_, PI(3,4,5)P_3_, and their kinases and phosphatases studied under the same signaling conditions.

The nuclear production of PI(3,4,5)P_3_ reflects the activities of various lipid kinases acting locally, and like PI(4,5)P_2_-binding proteins (Lewis et al., 2011), factors bound to PI(3,4,5)P_3_ may spatially and temporally alter the activities of the lipid kinases and downstream signaling (Tanaka et al., 1999). Accumulating evidence supports the notion that nuclear lipid kinases either exhibit signal-dependent translocation from the cytoplasmic compartment or are native to the nucleus, where their focal distribution and activities are regulated by various signals (Neri et al., 1994; Banfic et al., 2009; Kumar et al., 2011). So far, class I and II PI3Ks and inositol polyphosphate multikinase (IPMK)/Ipk2 activities have been observed within the nucleus.

Among the four class I PI3Ks (α, β, γ, and δ) that are implicated in cancer (Fruman and Rommel, 2014), the p110β catalytic isoform has been found in the nucleus (Kumar et al., 2011), where it may regulate S-phase progression (Marqués et al., 2008), DNA replication (Marqués et al., 2009), and DNA double strand break (DSB) repair (Kumar et al., 2010). It was demonstrated that only the nuclear and not the cytosolic pool of p110β was essential for cell survival in mouse embryonic fibroblasts (MEFs), and that the nuclear localization of p110β is mediated by the nuclear localization signal (NLS)-containing C2 domain (Kumar et al., 2011). This is consistent with previous findings that mice deficient in the p110β encoding gene PIK3CB are embryonic lethal before E3 even at the blastocyst stage (Bi et al., 2002), days earlier than deficiency of the gene encoding p110α (PIK3CA), which results in embryonic lethality around E10 (Bi et al., 1999; Foukas et al., 2006). The embryonic lethality of p110α and p110β deficiencies indicates the importance of PI3K in cell growth and embryonic development, possibly through the generation of distinct pools of PI(3,4,5)P_3_ required for the activation of downstream signals necessary for cell survival. It was found that only dual inhibition of p110α and p110β was sufficient to induce tumor regression of BT474 and MCF7 xenografts and prevent partial restoration of PI(3,4,5)P_3_ and phospho-Akt in HER2-amplified cell lines (Costa et al., 2015; Schwartz et al., 2015). These findings suggest that p110α and p110β have compensatory functions, where inhibition of one isoform initiates a feedback mechanism to activate the other. An earlier study indicated a similar compensatory phenomenon where despite p110α and p110δ contributing >90% of PI3K activity, only upon p110β inhibition was there a decrease in proliferation in p110α- and p110δ-mutant hematopoietic cells (Foukas et al., 2010). Furthermore, in MEFs, which mainly express p110α and p110β, ablation of both isoforms was required to reduce proliferation as a small fraction of total PI3K activity appeared sufficient to sustain cell viability (Foukas et al., 2010). Further investigation into nuclear p110β and its functions, apart from inducing Akt phosphorylation, may provide valuable insight into therapeutics targeting the p110 isoforms.

Class II PI3K-C2α was observed at nuclear speckles, implying a role in mRNA transcriptional regulation (Didichenko and Thelen, 2001). Indeed, speckle localization of PI3K-C2α correlates well with splicing factors depending on the transcriptional activities and signaling status of the cell (Didichenko and Thelen, 2001). It seems that the speckle-localized PI3K-C2α can be phosphorylation-modified with no impact on its catalytic activity during transcription inhibition, indicating non-canonical roles of PI3K-C2α within the nucleus (Didichenko and Thelen, 2001). PI3K-C2β was also found in the nuclear envelope, where tyrosine phosphorylation induced its lipid kinase activity for intranuclear PtdIns 3-phosphate (PI3P) generation (Visnjic et al., 2002), as well as in the nuclear matrix, where it can be proteolytically cleaved at the C2 domain for activation and local production of PI3P and to a lesser extent PtdIns 3,4-bisphosphate [PI(3,4)P_2_] (Sindic et al., 2006). Interestingly, the C2 domain of PI3K-C2β, which contributes to phospholipid binding and negative regulation of the catalytic activity, contains a nuclear localization motif that is required for PI3K-C2β nuclear matrix translocation stimulated by epidermal growth factor (EGF) (Arcaro et al., 1998; Banfic et al., 2009). Nuclear PI3K-C2β has potential roles in G_2_/M phase of cell cycle and growth regulation (Visnjic et al., 2003).

Similar to PI kinases which act on inositol rings bound to acyl chains, inositol kinases, such as IPMK, phosphorylate inositol rings without lipid tails to generate inositol 1,4,5,6/1,3,4,6-tetrakisphosphate (IP_4_), inositol 1,3,4,5,6-pentakisphosphate (IP_5_), and diphosphorylinositol tetrakisphosphate (PP-IP_4_) from inositol 1,4,5-trisphosphate (IP_3_) (Odom et al., 2000; Shears, 2004). In addition to the role of IPMK as an inositol kinase, IPMK exhibited wortmannin-insensitive and Akt signaling-independent phosphoinositol 3-phosphate kinase activity within the mammalian cell nucleus that out-performed nuclear PI3K for PI(3,4,5)P_3_ production (Resnick et al., 2005). Moreover, recent data suggest that IPMK enhances the transcriptional activity of the nuclear receptor steroidogenic factor 1 (SF-1)/NR5A1 by phosphorylating the solvent-exposed head group of its bound ligand, PI(4,5)P_2_(Blind et al., 2012). Phosphorylation of SF-1/PI(4,5)P_2_ generates SF-1/PI(3,4,5)P_3_ which induces formation of a novel protein-lipid interface by stabilizing the region around the ligand pocket (Blind et al., 2014). The protein-lipid interface allows SF-1 to interact with PI-binding proteins such as those containing PH-domains (Blind et al., 2014). It remains unclear how PIs are loaded into SF-1. However, SF-1 can be conjugated with SUMO-1 and thereby targeted to nuclear speckles (Chen et al., 2004). Sumoylation of SF-1, a plausible way of sequestering SF-1 from its nuclear targets, is a potential mechanism by which SF-1 is localized and loaded with ligand via direct uptake or by the action of phospholipid transport proteins (PLTPs). Another point requiring clarification is how the inhibition of SF-1 by sumoylation and phosphatase and tensin homolog (PTEN) dephosphorylation of SF-1-bound PI(3,4,5)P_3_ differ in their downstream effects. Additionally, because class I and class II PI3Ks and IPMK are all present within the nucleus, at nuclear speckles, and control gene expression, further work is needed to dissect the temporal, spatial, and functional assignments of these kinases in the nucleus.

Consistent with nuclear PI3K activities, Akt, a canonical downstream signaling effector of PI3K and an oncogene with critical roles in cell growth, has also been found within the nucleus. The nuclear presence of Akt can either be a result of native localization or translocation from the cytoplasm. Many targets of Akt can also be found within the nucleus such as the nucleolar phosphoprotein nucleophosmin (NPM)/B23, which dynamically shuttles between the nucleus and cytoplasm as well as from the nucleolus to the nucleoplasm in S-phase of the cell cycle (Borer et al., 1989; Itahana et al., 2003). Another Akt effector is mammalian target of rapamycin complex 1 (mTORC1). Raptor, a component of mTORC1, strongly associates with PtdIns 3,5-bisphosphate [PI(3,5)P_2_] and weakly with PI3P, presenting a mechanism by which these 3-phosphorylated PIs might regulate mTORC1 (Bridges et al., 2012). Interestingly, amino acid treatment increased the cellular levels of PI3P and PI(3,5)P_2_, which in turn induced localization of mTORC1 to late endosomes (Bridges et al., 2012; Jin et al., 2014). The presence of PI(3,5)P_2_ in the nucleus has not been documented. It is possible that PI(3,5)P_2_ has a role in nuclear function and signaling but has thus far evaded detection due to low abundance or requirements for specific signaling conditions.

Three Akt isoforms encoded by different genes have been identified in mammalian cells (Martelli et al., 2012). Akt1 and Akt2 mainly localize at the plasma membrane and cytosol, and may translocate to the nucleus after growth factor stimulation (Andjelkovic et al., 1997; Meier et al., 1997). Akt3, however, is predominantly localized within the nucleus and at the nuclear envelope (Santi and Lee, 2010). Given the nuclear production of PI(3,4)P_2_ and PI(3,4,5)P_3_ and their substrate binding capacities, the interaction of nuclear Akt(s) with these phospholipid messengers for activation in response to specific upstream signals must be a dynamic and well-orchestrated process. Interestingly, the 3-phosphoinositide-dependent protein kinase 1 (PDK1), which is known to phosphorylate and activate cytosolic Akt, is able to shuttle into the nucleus in a PI3K signaling- and nuclear PTEN-dependent manner, accumulating in the nucleus following insulin stimulation (Lim et al., 2003). It is not clear whether the nuclear localized PDK1 can activate Akt, and if the mechanism of the activation is similar to that in the cytoplasm. Further work to characterize the orchestration of cytosolic and nuclear activation of Akt by PDK1 is needed to connect or differentiate the two processes. Counteracting the nuclear localization of Akt, treatment of cells with extracellular ATP or statins induced a nuclear protein phosphatase- (PHLPP1/2, PP2A, and PTEN) and p110β-dependent nuclear Akt depletion (Mistafa et al., 2010; Ye et al., 2011). Balancing the kinase- and phosphatase-dependent and independent functions of nuclear kinases and phosphatases presents a significant challenge in the control of cell growth.

## Nuclear PI(3,4,5)P_3_ phosphatases

PI3K and Akt are kinases whose activities are antagonized by a variety of phosphatases. In addition to their canonical roles, phosphatases often have phosphate removal-independent functions. In the unique environment of the nucleus, phosphatase-dependent and -independent functions are essential for maintaining signaling equilibrium between activated and inactivated states.

The lipid phosphatase and tumor suppressor PTEN is localized to the cytosol as well as the nucleus (Chung and Eng, 2005; Liu et al., 2005). It was reported that PTEN sumoylation at K254 is critical for its nuclear retention which, like its phosphatase activity, is required for efficient genotoxic stress-induced DSB repair (Bassi et al., 2013). It was also demonstrated that nuclear localization without functional sumoylation of PTEN was not sufficient to recruit the recombinase RAD51 to sites of DNA damage to initiate DNA repair (Bassi et al., 2013). Additionally, PTEN was found to translocate to the nucleus following monoubiquitination (Trotman et al., 2007). These findings suggest the importance of posttranslational modification of PTEN for its nuclear functions. As described above, nuclear translocation of PTEN and other phosphatases was observed upon treatment with statins and extracellular ATP in insulin-stimulated A549 cells, where the phosphatases associated with phosphorylated Akt and facilitated nuclear Akt depletion (Mistafa et al., 2010). Under these circumstances, activation of the phosphatases coincided with a rapid nuclear translocation of proliferating cell nuclear antigen (PCNA), association of PCNA and p21^*Cip*1^, and cyclin D1 degradation (Mistafa et al., 2010). These changes induce cell cycle arrest in response to an absence of nuclear Akt, reiterating the importance of Akt localization within the nucleus.

Nuclear PTEN interacts with anaphase-promoting complex/cyclosome (APC/C) and its substrate-specific activator, CDH1, to promote APC-CDH1-mediated degradation of cyclin B (Song et al., 2011). Importantly, it is nuclear localization of PTEN and not its phosphatase activity that accounts for regulation of the APC-CDH1 ubiquitination complex (Song et al., 2011; Choi et al., 2014). On the other hand, APC and CDH1 facilitate removal of chromatin-bound PTEN, an important step for mitotic exit (Choi et al., 2014). PTEN was shown to localize to centromeres where it interacted with the DNA-binding centromeric protein CENP-C, a protein crucial for chromosome stability and integrity (Shen et al., 2007). The interaction of PTEN with CENP-C is another phosphatase-independent function of PTEN. Most, if not all, of the phosphatase activities of PTEN have been assigned to counteracting accumulation of PI(3,4,5)P_3_ at the plasma membrane with no effect on nuclear PI(3,4,5)P_3_ despite the presence of PTEN in the nucleus (Lindsay et al., 2006). This raises the possibility that PTEN phosphatase function requires conditions absent from the nucleus. The apparent lack of PTEN phosphatase function and the presence of the SH2 domain containing inositol 5-phosphatase 2 (SHIP2) in the nucleus necessitates further investigation into the quenching of nuclear PI3K activities and PI(3,4,5)P_3_ generation-triggered downstream signaling.

SHIP2 was found to concentrate in the nucleus, at nuclear speckles, and in the cytoplasm (Elong Edimo et al., 2011). Nuclear SHIP2 interacts with the nuclear lamina proteins Lamin A/C and the PP2A regulatory subunit PR130B (Elong Edimo et al., 2011). Interestingly, the SHIP2-PR130B complex was shown to translocate to the plasma membrane following EGF stimulation (Zwaenepoel et al., 2010). Counter to the canonically antagonistic function of phosphatases to PI3K/Akt signaling, silencing SHIP2 was found to decrease activated nuclear Akt and reduce cell adhesion and migration (Prasad, 2009). This implicates SHIP2 as a tumor promoter, and indeed, SHIP2 was found overexpressed in 44 percent of examined clinical breast cancer specimens (Prasad et al., 2008). The oncogenic activity of SHIP2 may be attributable, in part, to the requirement of PI(3,4)P_2_ for full Akt activation at the plasma membrane (Franke et al., 1997; Scheid et al., 2002). Similarly, nuclear Akt may rely on the nuclear production of PI(3,4)P_2_ from PI(3,4,5)P_3_ by SHIP2 to maintain or achieve full activation. It remains unclear how Akt activation by PI(3,4)P_2_ and PI(3,4,5)P_3_ is balanced and how SHIP2 activity is coordinated with the activity of other phosphatases.

## Regulation of mRNA processing and export by the nuclear PI3K pathway

Nuclear speckles are dynamic clusters of interchromatin granules enriched in mRNA processing factors (Spector and Lamond, 2011). Active transcripts were found around the periphery of speckles throughout the nucleoplasm (Misteli and Spector, 1998). The nuclear speckle localization of PI(4,5)P_2_, its kinases, and PI3K isozymes suggest a role of the nuclear PI3K pathway in gene transcription and mRNA processing.

PI(4,5)P_2_ is the predominant source for PI(3,4,5)P_3_ generation within cells. Considerable evidence supports that this holds true for the nuclear compartment (Cocco et al., 1987; Divecha et al., 1991; Boronenkov et al., 1998). Among the PI(4,5)P_2_ generating enzymes, only phosphatidylinositol phosphate kinase (PIPK) type I alpha (PIPKIα), type I gamma isoform 4 (PIPKIγi4), type II alpha (PIPKIIα), and type II beta (PIPKIIβ) have been identified within the nucleus with PIPKIα localizing to nuclear speckles, where it is involved in mRNA 3′-end processing (Boronenkov et al., 1998; Ciruela et al., 2000; Mellman et al., 2008; Schill and Anderson, 2009; Li et al., 2012).

Though PI(3,4,5)P_3_ has not been detected at nuclear speckles, its phosphatase SHIP2 was identified at nuclear speckles during mitosis when phosphorylated on S132 (Déléris et al., 2003; Elong Edimo et al., 2011). Consistent with its phosphatase activity, down-regulation of SHIP2 induced PI(3,4,5)P_3_ accumulation and Akt phosphorylation (Elong Edimo et al., 2011). These results implicate the presence of PI3K activity at the active sites of mRNA processing. This is supported by findings showing that PI3K-C2α colocalizes with mRNA splicing factors at speckles in a transcriptional activity-dependent manner (Didichenko and Thelen, 2001) and that nuclear IPMK's PI(3,4,5)P_3_-kinase activity is involved in transcriptional regulation (Odom et al., 2000; Resnick et al., 2005). In addition to acting as a PI(3,4,5)P_3_ phosphatase, SHIP2 can interact with and dephosphorylate PI(4,5)P_2_ (Elong Edimo et al., 2011). This may potentially influence the activities of the speckle PI(4,5)P_2_-regulated nuclear poly (A) polymerase Star-PAP (Mellman et al., 2008) and PKCδ required for Star-PAP control of DNA-damage response gene expression (Li et al., 2012). The addition of a poly (A) tail to pre-mRNA by poly (A) polymerases is important for stabilizing the transcript and identifying it for nuclear exportation (Fuke and Ohno, 2008). Although absent from nuclear speckles, a nuclear PI(3,4,5)P_3_ pool was found within the nuclear matrix that may be involved in the regulation of active transcription and mRNA processing (Lindsay et al., 2006). This PI(3,4,5)P_3_ pool is insensitive to nuclear PTEN expression (Lindsay et al., 2006). Although PTEN has not been assigned to any particular nuclear structure, a rapid perinuclear accumulation of PTEN has been observed upon atorvastatin treatment (Mistafa et al., 2010). These studies suggest the intriguing concept that different phosphotidylinositol 3-phosphate kinase and phosphatase activities are required for nuclear speckle- and nuclear matrix-targeted transcriptional and post-transcriptional processes.

Post-transcriptional processing of mRNA is required for the stability and export of the message for translation. It has been shown that the PI3K/Akt pathway regulates mRNA export by mediating the assembly of the transcription-export (TREX) complex at the 5′-end of mRNA and the exon junction complex (EJC) (Quaresma et al., 2013). As a key adaptor protein within TREX, the mRNA export factor Aly was shown to be regulated by nuclear PI3K activity through phosphorylation by nuclear Akt and association with PI(4,5)P_2_ and PI(3,4,5)P_3_ (Okada et al., 2008). Both phosphorylation by Akt and phosphoinositide association were required for Aly regulation of mRNA export and cell proliferation (Okada et al., 2008). Remarkably, it was demonstrated that selective export of gene transcripts, including that of RAD51, are regulated by the pathway, suggesting a signaling-guided mRNA export model (Okada et al., 2008; Wickramasinghe et al., 2013). Supporting this concept, the generation of PI(3,4,5)P_3_ by the PI3K-like activity of IPMK appears to be required for the sequence-based selective export of mRNAs encoding proteins involved in DNA repair by homologous recombination (HR) (Wickramasinghe et al., 2013). Nuclear lipid kinase regulation of mRNA processing and export bridges the outside-in and inside-out signaling mechanisms crucial for adaptive protein synthesis and cell survival.

## Ribosome biogenesis

**Figure 2:**
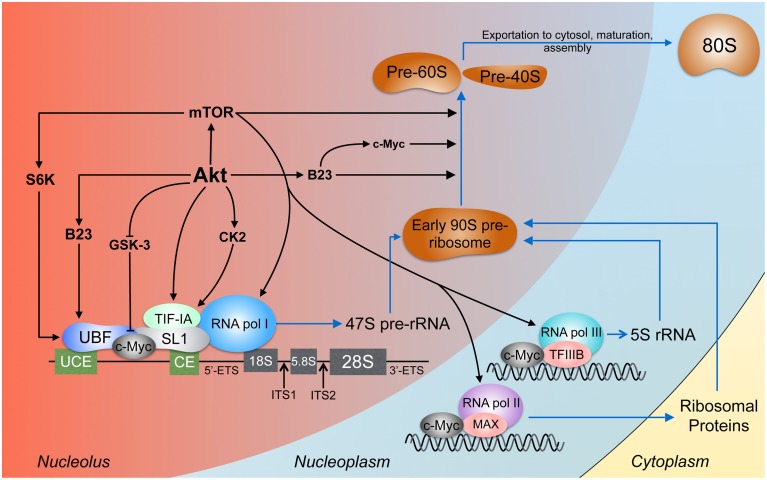
**Akt signaling in ribosome biogenesis**. Ribosome biogenesis occurs largely in the nucleolus. The pre-initiation complex is composed, among other proteins, of RNA pol I, UBF, which binds the upstream control element (UCE) and the core element (CE), and SL1, which binds the CE (Knutson and Hahn, 2013). The products of RNA pol I and II and the ribosomal proteins conjoin to form the early 90S pre-ribosome which is processed to the pre-60S and pre-40S subunits (Grandi et al., 2002; Tschochner and Hurt, 2003). Akt activates rDNA transcription by stabilizing c-Myc, B23, and TIF-IA and through CK2 which also acts on TIF-IA. TIF-IA interacts directly with SL1 and RNA pol I, enhancing rDNA transcription (Miller et al., 2001). Through S6 kinase-1 (S6K-1), mTOR facilitates the interaction between UBF and SL1 (Hannan et al., 2003). mTOR can bind 5S rDNA promoters (Shor et al., 2010). The expression of rDNA transcribed by RNA pol I, II, and III is enhanced by c-Myc (Eilers and Eisenman, 2008) through direct rDNA binding and association with SL1, MAX (Amati et al., 1993), and TFIIIB (Gomez-Roman et al., 2003). Processing of the pre-rRNA transcripts involves Akt, c-Myc (Schlosser et al., 2003), mTORC1 (Iadevaia et al., 2012), and B23 (Maden, 1990; Savkur and Olson, 1998).

Ribosome biogenesis is the process by which cells synthesize and assemble components of the translational machinery (Figure 2). The intimate connection between ribosome biogenesis and tumorigenesis is evident through the increased incidence of cancer and risk of neoplasia when ribosome synthesis is altered or upregulated (Loreni et al., 2014). With approximately 400 ribosomal DNA (rDNA) tandem repeats distributed across five chromosomes in humans (Henderson et al., 1972; Birch and Zomerdijk, 2008) and more than 60% of total cellular transcription devoted to ribosome biosynthesis (Warner, 1999), it is not surprising that this process is highly regulated and often dysfunctional in cancer. The nucleus, specifically the nucleolus, lies at the heart of the energetically demanding and complex synthesis of ribosomes.

Ribosome biosynthesis is initiated in the nucleolus where ribosomal RNA (rRNA) is synthesized from rDNA, a process requiring assembly of preinitiation complexes (PICs) for synthesis of the primary 47S pre-rRNA transcript. The PIC is composed of selectivity factor 1 (SL-1), upstream binding factor (UBF), the RNA polymerase I (RNA pol I) transcription factor RRN3/TIF-IA, RNA pol I and additional cofactors (Leary and Huang, 2001). Though nucleolar localization of PI3K has not been defined, the p110β and p85 subunits of PI3K were found to interact with insulin receptor substrate-1 (IRS-1) and UBF within the nucleus upon insulin-like growth factor-1 (IGF-1) stimulation, enhancing rRNA synthesis (Drakas et al., 2004). This study supports another observation that IGF-1- and nutrient-induced RNA pol I activation was completely dependent on PI3K activity and modulated by mTOR (James and Zomerdijk, 2004). These findings provide a mechanism by which nutrient status and growth factors regulate ribosome biogenesis through PI3K and MAPK signaling acting on RNA Pol I, further establishing the roles of the PI3K and MAPK pathways in cell growth and proliferation.

In addition to RNA pol I regulation of rRNA production, ribosome biogenesis also involves RNA pol II and III for ribosomal protein-encoding pre-mRNA synthesis and 5S rRNA generation, respectively. As a cellular biosensor for energy, nutrient, and growth status, mTOR has been implicated in multiple levels of regulation of ribosome biogenesis including modulation of the activities of RNA pol I, II, and III (Mayer and Grummt, 2006). Despite its central role in ribosome biogenesis, recent studies in lymphoma and leukemic cells have shown that inhibition of mTOR accounts for only part of the effect on rRNA synthesis observed during Akt inhibition, suggesting that Akt-mediated regulation of ribosome biogenesis occurs through mTOR-dependent and -independent pathways (Chan et al., 2011; Nguyen and Mitchell, 2013).

One of the roles of nuclear Akt in ribosome biogenesis involves regulation of rRNA synthesis. Akt stabilizes and, through casein kinase 2α (CK2α), activates transcription initiation factor-I (TIF-I), thus enhancing RNA pol I tethering to rDNA promoters (Nguyen and Mitchell, 2013). Akt also interacts with nuclear PI(3,4,5)P_3_ and B23, interactions that likely involve nuclear PI3K activities (Ahn et al., 2005; Lee et al., 2008; Kwon et al., 2010). B23, protected from proteolytic cleavage by nuclear Akt (Lee et al., 2008), is essential for the nucleolar localization and regulation of rDNA transcription by c-Myc (Li and Hann, 2013), a universal transcription factor and proto-oncoprotein (Dang, 2012; Nie et al., 2012).

The contribution of c-Myc to malignancy is well-established and, in part, attributable to the promotion of ribosome biogenesis (Tansey, 2014). Among other roles, nuclear c-Myc controls the transcription of UBF, directly binds rDNA in the nucleolus, mediates assembly of SL1 on rDNA promoters, facilitates UBF and TATA-binding protein (TBP) assembly into the PIC, and increases histone acetylation and chromatin accessibility (Poortinga et al., 2004; Grandori et al., 2005). The interaction of c-Myc with rDNA, found to occur at intergenic spacer regions (IGSs), is important for epigenetically non-silenced and promoter-hypomethylated rDNA attachment to the nuclear matrix for transcriptional activation (Littlewood et al., 1995; Shiue et al., 2014). Matrix attachment of rDNA occurs in response to growth factor stimulation, a signal that may be relayed to c-Myc through the PI3K pathway.

The purview and regulation of c-Myc are vast and fascinatingly complex and are known to involve PI3K signaling through Akt (Thomas and Tansey, 2011; Spender and Inman, 2014). In the case of constitutive PI3K signaling, among other c-Myc activating events (Taub et al., 1982; Bahram et al., 2000; Yamamura et al., 2012), c-Myc persists in the nucleus. Normally, c-Myc has a short half-life of 15–20 min due to rapid proteolytic degradation initiated through phosphorylation of residue T58 by glycogen synthase kinase-3 (GSK-3) (Gregory et al., 2003). The importance of c-Myc, and specifically T58, was shown in c-Myc^*T*58*A*^ knock-in mice. c-Myc^*T*58*A*^ mice exhibited enhanced mammary gland density, hyperplastic foci, cellular dysplasia, and mammary carcinomas relative to wild-type mice, indicating increased genomic instability and apoptotic suppression (Wang, 2011). This is consistent with previous findings that inhibition of T58 phosphorylation enhances the transforming activity of c-Myc by concomitantly decreasing c-Myc proteolysis and apoptotic potential (Conzen et al., 2000). Akt increases the half-life of c-Myc through GSK-3 by at least two means. First, Akt phosphorylates GSK-3, rendering it catalytically inactive and unable to phosphorylate c-Myc on T58 (Wang et al., 1994). Second, Akt facilitates nuclear export of GSK-3 by promoting its interaction with the chaperone protein Frat (Bechard et al., 2012). Since GSK-3 lacks a nuclear export signal (NES), interaction with Frat, which possesses a Crm1-dependent leucine-rich NES, represents a mechanism by which GSK-3 is separated from its nuclear targets and exported from the nucleus (Franca-Koh et al., 2002). Interestingly, Akt-independent regulation of GSK-3 phosphorylation was identified in PI3Kγ knockout mice. The kinase-independent activity of PI3Kγ inhibited the interaction between the phosphatase PP2A and its methyltransferase, PPMT-1, required for GSK-3 dephosphorylation and activation (Mohan et al., 2013). To date, no definitive evidence has been presented addressing a direct interaction between Akt and GSK-3 in the nucleus. Acting independent of and with the transcription factor c-Myc, Akt directly and indirectly coordinates and promotes various aspects of ribosome biogenesis.

It was known as early as the nineteenth century that cancer cells have irregularly shaped and enlarged nucleoli (Giuseppe, 1896). We now know that these structural changes are associated with cellular stress and often, disruptions in ribosome biogenesis. Defects in nucleolar integrity result in release of ribosomal proteins to the nucleoplasm, where ribosomal proteins like RPS14 can inactivate the E3-ubiquitin ligase activity of MDM2, stabilizing p53 and thereby inducing cell cycle arrest (Zhou et al., 2013). RPS14 was also found to inhibit the transcriptional activity of c-Myc by preventing recruitment of c-Myc and transformation-transcription domain-associated protein (TRRAP) to c-Myc target gene promoters (Zhou et al., 2013). TRRAP is a PI3K-related pseudokinase possessing a domain that is highly homologous to the kinase domain of p110 subunits of PI3K but lacks the capacity to phosphorylate substrates (McMahon et al., 1998). It is possible that TRRAP has a scaffolding role resembling that of PI3K, and serves to stabilize protein complexes involved in ribosomal biogenesis. Interestingly, TRRAP deletion significantly reduced the expression of ribosomal proteins (Tapias et al., 2014). Additionally, two other ribosomal proteins, RPL5 and RPL11, were found to cooperate in guiding the RNA-induced silencing complex (RISC) to c-Myc mRNA while RPL11 also decreased histone H4 acetylation at c-Myc target gene promoters, effectively inhibiting c-Myc activity (Dai et al., 2007; Liao et al., 2014). It is clear ribosomal proteins have crucial cellular functions as befits their early emergence in evolution. The abundance and dispersal of ribosomal protein-coding genes throughout the genome constitutes a unique sensor by which cells can detect genomic aberrations (Kim et al., 2014). Genomic instability will often disrupt the stoichiometric ratio of ribosomal proteins to rRNA or cause the loss of nucleolar integrity, triggering p53-dependent and -independent downstream effects (Alt et al., 2005). Cancerous cells, often exhibiting aneuploidy, must avoid triggering these sensors. Exploitation of the activities of ribosomal proteins for therapeutic intervention may someday prove a viable method of cancer treatment. However, despite the role of ribosome biogenesis in satisfying the enhanced biosynthetic demand of cancerous cells, the degree to which deregulation of ribosome biogenesis is causative of or auxiliary to tumorigenesis is unclear.

## DNA replication and damage repair

Genomic integrity is under constant threat from both endogenous and exogenous factors. Replication fidelity and repair of damaged DNA ensures correct genetic information is carried over during cell division and proliferation. These processes are critical to genomic integrity and even slight deviations can result in age-associated diseases and cancer (Hoeijmakers, 2001). The PI3K signaling pathway has been implicated in many processes of cell cycle regulation including DNA replication and damage repair. Moreover, the various PI3K isoforms seem to be differentially involved in cell cycle regulation. For instance, p110α is activated at G_1_-phase entry and promotes PI(3,4,5)P_3_ and protein synthesis and gene expression whereas p110β activity peaks in S-phase and regulates DNA synthesis and protein activities for cytokinesis (Silio et al., 2012).

A central component of DNA replication and repair is PCNA, a eukaryotic sliding clamp protein. Among its numerous functions, PCNA triggers displacement of Polα/primase and acts as a loading platform for the processive DNA polymerases (Maga et al., 2000). PCNA requires the ATPase activity of the clamp loader, replication factor C (RFC), to open and encircle double-stranded DNA (Indiani and O'Donnell, 2006). Through a kinase-independent function, p110β was found to interact with RFC1, a subunit of the RFC complex, and thereby promote loading of PCNA onto chromatin (Redondo-Muñoz et al., 2013). It is interesting to note that p110β regulates PCNA loading through both kinase-dependent and -independent activities as phosphorylation of the cell cycle inhibitor p21^Cip1^ on T145 releases PCNA from its suppressive binding to p21^Cip1^ (Marqués et al., 2009). Depletion of p110β with RNA interference (RNAi) increased the expression levels of p21^Cip1^ and its association with PCNA, and impaired PCNA-RFC association and loading onto chromatin (Marqués et al., 2009; Redondo-Muñoz et al., 2013). The interaction of PCNA and p21^Cip1^, occurring through the same domain as the PCNA-DNA pol δ interaction, negatively regulates S-phase progression (Cazzalini et al., 2003). The defects in S-phase progression induced by p110β knockdown can be recovered by expression of a phosphomimetic p21^Cip1^ mutant (Marqués et al., 2009), emphasizing the requirement for an active PI3K signaling cascade in DNA replication.

Among DNA damage lesions, the most detrimental to genomic integrity are DNA double-strand breaks (DSBs). Commencement of DSB repair begins with establishment of large protein complexes, referred to as foci, that contain DNA repair proteins (Paull et al., 2000). Found at DNA damage foci, p110β was required for the recruitment of Nijmegen breakage syndrome-associated gene product, Nibrin/NBS1, and PCNA to DSBs (Kumar et al., 2010). p110β-null MEFs exhibited spontaneous DSBs coincident with abnormal chromosome numbers and chromosome breaks (Kumar et al., 2010). p110β RNAi in NIH-3T3 cells and p110β deletion in MEFs rendered the cells unable to activate the G_2_/M checkpoint (Kumar et al., 2010). Consistent with a role in DNA replication, Akt has been implicated in DNA damage repair. The finding that nuclear Akt is phosphorylated at S473, normally targeted by mTORC2 (Li et al., 2007), much earlier than cytoplasmic Akt after irradiation in GM0719 cells (Boehme et al., 2008) indicates that DNA damage induces rapid Akt activation in the nucleus. Likewise, irradiation-induced Akt nuclear translocation and accumulation was observed, and Akt was found colocalized with DSB marker γH2AX at DNA break sites (Liu et al., 2014). These observations indicate the critical role of the nuclear p110β and Akt in the maintenance of genomic stability, the disruption of which is a hallmark of cancer (Negrini et al., 2010).

Nuclear PI3K regulation of the DNA damage response may be mediated by factors such as the PI3K enhancer (PIKE) and the proto-oncogene product c-Abl. The interaction of PIKE with nuclear PI3K stimulates the lipid kinase activity of PI3K (Ye et al., 2000) necessary to antagonize apoptosis (Ahn et al., 2004). The non-receptor tyrosine kinase c-Abl directly binds and phosphorylates p85 in response to γ-irradiation, thereby inhibiting PI3K activity (Yuan et al., 1997). Interestingly, this inhibitory role of c-Abl on PI3K activity contrasts with the PI3K-activating roles of the transforming Bcr-Abl and v-Abl variants, where an N-terminal myristoylation of the Abl proteins was found to be required to recruit PI3K to the plasma membrane for activation and generation of PI(3,4,5)P_3_ (Varticovski et al., 1991). This PI3K activation model more aptly applies to cytoplasmic membrane structures as the Bcr-Abl fusion protein is found exclusively in the cytoplasm and promotes apoptosis when entrapped in the nucleus (Vigneri and Wang, 2001; Dixon et al., 2009). However, since c-Abl is also present in the cell nucleus (Van Etten, 1999), it is of interest to determine the degree to which it modulates nuclear PI3K activity.

## Cell survival and tumorigenesis

The early embryonic lethality of p110β-null mice indicates the indispensability of p110β for cell survival, growth, and development (Bi et al., 2002). Supporting this, NIH-3T3 cells lacking p110β exhibited increased susceptibility to spontaneous and γ-radiation-induced apoptosis (Kumar et al., 2011). Introducing wild-type p110β but not a C2-NLS p110β mutant (targeting p110β to the cytoplasm) restored cell survival, reiterating the critical role of nuclear p110β in cell viability (Kumar et al., 2011). Interestingly, mice deficient in the catalytic activity of p110β (K805R), other than incomplete penetrance of embryonic lethality, survived normally, but were born smaller than wild-type controls and showed growth retardation (Ciraolo et al., 2008). Despite the lack of recurrent mutations within the p110β-encoding gene PIK3CB (Vanhaesebroeck et al., 2010), the catalytic activity of p110β, but not that of p110α, is responsible for active human epidermal growth factor receptor 2 (HER2)-induced mammary tumor cell growth and carcinogenesis (Ciraolo et al., 2008). Similarly, expression of constitutively active p110β in the prostate of mice induced intraepithelial neoplasia (Lee et al., 2010). In addition, p110β, but not p110α, seems to be responsible for prostate cancer development in the absence of PTEN (Jia et al., 2008). Furthermore, overexpression of p110β induced cell transformation in a MAPK/Erk- and Akt/mTOR-dependent manner (Denley et al., 2008), indicating cross talk between the two growth-regulating pathways during oncogenic transformation. The capacity of PI3K for kinase-dependent and -independent functions is not limited to p110β. The kinase activity of p110γ was found to regulate Akt and MAPK phosphorylation while its kinase-independent function controls cAMP and cGMP levels (Patrucco et al., 2004). These observations in mice and cancer cells suggest that both the kinase-dependent and -independent functions of p110β are required for cell growth and proliferation.

Nuclear PI3K and its upstream regulator PIKE were found to be necessary and sufficient to mediate the anti-apoptotic effect in growth factor-stimulated PC12, HEK-293, and HeLa cells by preventing DNA fragmentation initiated by a cell-free apoptosome (Ahn et al., 2004). In this study, addition of PI(3,4,5)P_3_ to cell nuclei mimicked the anti-apoptotic effect but required nuclear Akt (Ahn et al., 2004). The effect of PI(3,4,5)P_3_ on apoptotic prevention may be due to its control over the interaction between Akt and the nuclear PI(3,4,5)P_3_ receptor, B23 (Kwon et al., 2010). The Akt–B23 interaction enhances cell survival by preventing the caspase-3 dependent degradation of B23 (Lee et al., 2008). In this scenario, the interaction of Akt and B23 occurs between the N-terminal PH domain of Akt and the C-terminus of B23, and, importantly, requires Akt phosphorylation and nuclear translocation as well as activation triggered by growth factor stimulation (Lee et al., 2008). Interestingly, the kinase activity of Akt was not required for this interaction and a constitutively active Akt with phospho-mimetic mutations (T308D, S473D) strongly bound B23 (Lee et al., 2008), indicating that a phosphorylation-induced conformation change of Akt potentiates binding and determines substrate specificity. The stabilization of B23 by Akt is important as B23 directly interacts with c-Myc and stimulates c-Myc-induced hyperproliferation and transformation (Li et al., 2008). Just as Akt protects c-Myc from degradation by phosphorylating and excluding GSK-3 from the nucleus, facilitating ribosome biogenesis (Wang et al., 1994; Gregory et al., 2003; Bechard et al., 2012), Akt was shown to cooperate with c-Myc to regulate fibroblast proliferation and transformation through PI3K-dependent phosphorylation of the FOXO transcription factors (Brunet et al., 1999; Bouchard et al., 2004). Unphosphorylated FOXO proteins inhibit c-Myc-induced proliferation and transformation and repress formation of the transcription preinitiation complex at the promoter site of certain genes including cyclin D2 (Bouchard et al., 2004). Phosphorylation of FOXO by Akt occurs downstream of PI3K activation and triggers FOXO nuclear export thus derepressing multiple c-Myc target genes (Bouchard et al., 2004).

Extracellular ATP, atorvastatin, and a purinergic receptor P2X7 inhibitor were all shown to suppress cell growth by inducing rapid depletion of phosphorylated nuclear Akt, a process requiring nuclear localization of p110β (Ye et al., 2011). Nuclear depletion of Akt was preceded by the nuclear localization of PTEN, PHLPP, and calcineurin, and could be prevented by inhibition of the scaffolding immunophilin FK506-binding protein 51 (FKBP51) or calcineurin (Mistafa et al., 2010). In these scenarios, p110β can act as a scaffold between Akt and the phosphatases PTEN, PP2A, and PHLPP1/2 (Gao et al., 2005; Brognard et al., 2007; Mistafa et al., 2010; Kumar et al., 2011; Ye et al., 2011). This may help to explain the function of the overexpression of p85β, a regulatory subunit of PI3K, in cancers, where p85β and p110β cooperate to retain activated nuclear Akt, facilitating cell survival, cell cycle progression, and cell growth. It is plausible that the requirement of p110β for depletion of nuclear Akt is a result of its scaffolding role in the FKBP51-mediated interaction between Akt and its phosphatase PHLPP, promoting dephosphorylation of nuclear Akt by PHLPP (Pei et al., 2009). The recently reported feedback inhibition of PHLPP degradation by Akt-mediated GSK-3β inactivation adds complexity to the evolving paradigm (Li et al., 2009). It remains unclear if p110β-mediated nuclear depletion of Akt is a mechanism for excess Akt sequestration and if so, how this is related to nuclear PI3K activity-induced Akt activation. Additionally, it is not known whether nuclear Akt depletion is dependent on Akt dephosphorylation by PHLPPs.

A variety of human cancers exhibit increased nuclear accumulation of Akt, a phenomenon that varies over the progression of the pathological lesion (Ye et al., 2011). Copy number changes of the genes encoding all three Akt isoforms were observed in estrogen receptor (ER)-positive breast carcinomas (Kirkegaard et al., 2010). Amplification of Akt3 and deletions of Akt1 and Akt2 were observed in breast carcinomas while Akt1 amplification was the only observed Akt copy number alteration in prostate carcinomas (Kirkegaard et al., 2010). In a mutational analysis for polymorphism, Akt2 mutations were identified in patient samples with gastric or lung carcinomas (Soung et al., 2006). Overexpression of Akt2 at the post-transcriptional level was found in oral squamous cell carcinomas, where Akt3 mRNA was expressed at low levels (Iamaroon and Krisanaprakornkit, 2009). These findings collectively suggest a differential expression and regulatory pattern of the Akt isoforms in different cancers, likely linked to the nuclear activities of Akt.

The Akt activating factor PDK1, which phosphorylates Akt at T308 in the cytoplasm, was found to shuttle between the cytoplasm and the nucleus (Lim et al., 2003). Nuclear-localized PDK1 was found to induce the formation of solid tumors and correlated with an increase in phosphorylated nuclear Akt, which suppressed FOXO3A-mediated p27^kip1^ expression (Kikani et al., 2012). While both cytoplasmic and nuclear PDK1 are able to induce tumor formation, nuclear PDK1 is associated with higher-risk tumors than cytoplasmic PDK1 (Kikani et al., 2012). These findings indicate important roles of nuclear PDK1 in oncogenic transformation and tumorigenesis, and warrant further investigation into the isoform-specific activation of nuclear Akts by PDK1.

## Prospects of nuclear PI3K signaling

**Figure 3:**
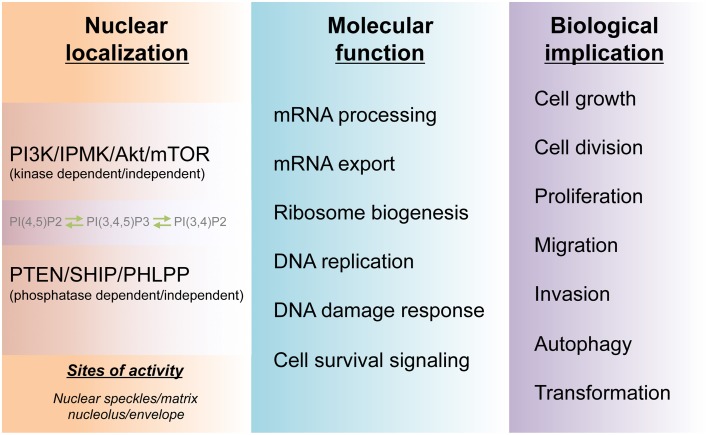
**Molecular and biological functions of the nuclear PI3K pathway**. The cell nucleus harbors a PI3K pathway that is functionally distinct from that of cytoplasm. The non-membranous localization of the nuclear phosphoinositides PI(4,5)P_2_, PI(3,4)P_2_, and PI(3,4,5)P_3_ and their kinases as well as phosphatases form hubs for channeling divergent signaling to downstream molecular functions in the nucleus. These nuclear activities, in turn, guide cell survival and many physio-pathological processes including tumorigenesis.

It has been established that PI3K signaling plays essential roles in cell growth and tumorigenesis. Both basic and clinical research data suggest that the different PI3K and Akt isoforms as well as their counteracting phosphatases and effector proteins are spatially and temporally regulated within cells (Figure 3). These kinases are expressed and regulated at different levels in different cancer cells and are often genetically modified during cell transformation. The complexity of this signaling nexus does follow a pattern as subcellular targeting of specific PI3K and Akt isoforms determines many if not all of the downstream signaling events.

Evidence for additional avenues of transduction beyond the canonical PI3K/Akt signaling cascade has emerged. First, PI3K/IPMK may signal to downstream effectors without activating Akt (Resnick et al., 2005; Mohan et al., 2013). Other signaling pathways such as the MAPK/Erk cascade may crosstalk with and/or be activated/deactivated through PI3K/Akt signaling and inhibition (Mendoza et al., 2011). Consequently, combinational inhibition of the signaling pathways in the clinical treatment of cancer patients has been introduced. These methods are promising in increasing the efficacy of treatments but, at the same time, may cause complications (i.e., increased toxicity and chemoresistance) at both the molecular and systemic levels (McCubrey et al., 2012; Saini et al., 2013). It must also be considered that PI3K, PTEN, and likely other proteins in the signaling cascades have kinase- or phosphatase-independent functions (Lindsay et al., 2006; Dou et al., 2010; Ye et al., 2011). Secondly, there are additional upstream activating protein kinases that can induce cell proliferation and malignancy through Akt independent of PI3K (Mahajan and Mahajan, 2012). Third, as for many oncogenes in certain types of cancers, PI3K or Akt hyperactivation requires additional signaling inputs, such as PTEN loss, to induce tumor formation (Jia et al., 2008). Fourth, mutations in different domains of a kinase in the cascade may alter the conformation of the protein in a way that requires specific co-factors for activation, yielding distinct cancer phenotypes (Klarenbeek et al., 2013). Notably, clinical observations are not always consistent with the traditional views of PI3K/Akt activity. For example, nuclear localization of activated Akt was associated with long-term survival of breast cancer patients especially within the ERα+/PR+ subgroup when compared with nuclear phosphorylated Akt-negative patients (Badve et al., 2010). Additional work on the non-canonical roles of the PI3K signaling pathway will be necessary for the development of more efficacious cancer treatments. In contrast, downregulation of Akt isoforms sensitizes chemoresistant endometrial carcinoma cells to chemotherapeutic drugs (Girouard et al., 2013). It is intriguing that this seems to be an isoform specific effect since constitutive Akt1 or Akt2 expression led to increased resistance to apoptosis (Girouard et al., 2013), suggesting a possibility of selective targeting of signaling molecules to increase chemo/radiation-sensitivity.

Encouraging progress has been made in defining nuclear PI3K/Akt functions in cell growth and tumorigenesis. Nevertheless, many questions remain to be answered: To what degree do the nuclear and cytosolic PI3K cascades complement or interfere with one another during tumorigenesis? What other nuclear signaling cascades coordinate and cross-regulate with the nuclear PI3K pathway? How does the cell differentially regulate the activities of highly homologous isoforms present in the same subcellular compartment, as in the case of the Akt isoforms in the nucleus? Similarly, how are the kinase- or phosphatase-dependent and -independent functions of a single isoform separately regulated? How are the activities of specific kinases and phosphatases balanced during tumorigenesis and pharmacological treatment? Elucidating the nuclear PI3K pathway will complement cytoplasmic findings and contribute to the development of more effective clinical interventions against cancer.

### Conflict of interest statement

The authors declare that the research was conducted in the absence of any commercial or financial relationships that could be construed as a potential conflict of interest.
